# Sex and age differences in cortisol levels during glucagon stimulation test in children

**DOI:** 10.1186/s12887-025-05784-5

**Published:** 2025-05-31

**Authors:** Camilla Borghammar, Johan Svensson, Anders Tidblad, Maria Elfving

**Affiliations:** 1 Department of Clinical Sciences, Paediatric Endocrinology, Lund University, Skåne University Hospital, Lund, Sweden; 2 Department of Women’s and Children’s Health, Division of Pediatric Endocrinology, Karolinska Institutet, Karolinska University Hospital, Solna, Sweden

**Keywords:** Cortisol, Glucagon test, Child, Childhood, Sex-differences

## Abstract

**Background:**

Previous studies of glucagon stimulation test (GST) in children have shown variable results regarding the utility and reliability of the cortisol response to this test and its correlation with clinical parameters. The aim of this study was to assess cortisol levels at GST and to evaluate how clinical parameters, such as age, sex, pubertal status and Body Mass Index (BMI), correlate to cortisol levels in children.

**Methods:**

A retrospective study of children evaluated for short stature with the GST. Cortisol, glucose and growth hormone (GH) levels at GST, as well as clinical parameters (age, sex, pubertal status, BMI), were collected from medical records. A peak cortisol of *≥* 450 nmol/L was used as a cut-off indicative of a sufficient response. Non-parametric tests were applied in the statistical analysis, and linear regression was used to examine factors affecting cortisol max at the GST.

**Results:**

In total, 171 children were included; median age 7.8 years (1.0–18.0), 60 (35.1%) female, 23 (13.5%) pubertal. Of all children, 145 (84.8%) achieved a peak cortisol *≥* 450 nmol/L. There was a negative correlation between peak cortisol levels and age (Spearman’s rho − 0.26, p = < 0.001). Peak cortisol levels were higher in females vs. males: 667.5 nmol/L (range 400–995) vs. 602 nmol/L (range 202–1008), *p* = 0.005. A higher number of boys than girls did not reach the cortisol cut-off value of 450 nmol/L (*p* = 0.022). The difference in maximum stimulated cortisol levels between the sexes remained after adjusting for age with a linear regression model (β (95% CI) 65.3 (15.9–114.6), *p* = 0.01).

**Conclusion:**

GST is a reliable test of the hypothalamic-pituitary-adrenal (HPA) axis in children. Girls and younger children had higher peak cortisol at GST. The results support a need for sex- and age-dependent reference values for cortisol.

**Supplementary Information:**

The online version contains supplementary material available at 10.1186/s12887-025-05784-5.

## Background

Undiagnosed adrenal insufficiency can cause a life-threatening cardiovascular collapse [1]. To avoid this, there is a need for safe and reliable tests to assess the hypothalamic-pituitary-adrenal (HPA) axis in children.

The glucagon stimulation test (GST) and adrenocorticotropic hormone (ACTH) stimulation test are pharmacological stimulation tests that can be used to assess adrenal cortisol production [2, 3, 4, 5]. According to Endocrine Society Guidelines, the ACTH stimulation test is primarily recommended to evaluate the HPA axis in adults [6]. However, the GST may have advantages in diagnosing secondary adrenal insufficiency, since it evaluates the HPA axis, in contrast to the ACTH stimulation test, which evaluates the adrenal response to synthetic ACTH [2, 7]. Additionally, the GST simultaneously assesses the growth hormone (GH) axis [7].

The exact mechanism of the GST on the HPA and GH axis is not completely understood. It has been suggested that it is linked to glucagon-induced fluctuations in glucose levels, as well as glucagon-induced arginine vasopressin/copeptin or noradrenalin-related activation of the HPA axis [8, 9, 10].

There is no universally accepted cut-off for normal cortisol response to GST in children, and different levels have been suggested [3, 4, 5, 11, 12]. In a study of 290 children, Böttner et al. recommended a cut-off value of 450 nmol/L for cortisol at GST [5]. This is the same cut-off that is recommended for adults at standard ACTH stimulation test and using the Roche Cortisol II method, according to a consensus from the Swedish Endocrinological Association, Swedish Society for Clinical Chemistry and Equalis (2017) [13].

Previous studies have investigated the GST cortisol response in children (suppl. Table 1) [3, 4, 5, 11, 12, 14, 15, 16]. It has been suggested that clinical parameters, such as age, sex, Body Mass Index (BMI) and pubertal status affect the cortisol response. However, as described in supplemental Table 1, previous studies have shown divergent results of the correlation between cortisol levels and clinical parameters at GST in children [4, 5, 12, 14, 15, 16]. Some studies found no correlations for sex or for age and stimulated cortisol levels, while other studies found a negative correlation between age and peak cortisol [4, 5, 12, 14, 15, 16]. In addition, some studies reported sex-related differences in peak cortisol at GST, with higher levels for girls [12, 14, 15].

The aim of this study was to assess cortisol levels at GST and to evaluate how clinical parameters (age, sex, pubertal status, BMI) correlate to cortisol levels in children with short stature or low growth velocity.

## Methods

### Patient inclusion

In this retrospective study, included patients were 0-18.99 years old and had undergone a GST between 2018 and 2023 at Karolinska University Hospital, Stockholm, Sweden or between 2020 and 2023 at Skåne University Hospital, Malmö, Sweden. Arginine stimulation test (AST) and GST were used to evaluate GH levels and possible GHD in children with short stature and/or low growth velocity. Cortisol levels were also tested. First, an AST was performed, and if the GH level at testing was lower than the cut-off level for the GH method used, the child also underwent a GST, most often the following day. The same protocol was used at both centres.

Patient exclusion criteria were: *≥*19 years old, no available medical records, test incorrectly coded as GST, more than one missing cortisol value after administration of glucagon. At inclusion of patients, we used KVÅ codes (procedure code numbers) to search hospital registries. At Karolinska University Hospital we searched hospital registries for GST (KVÅ code: AB013). In this search, we identified 149 patients, of whom one was excluded because of age > 18.99 years. At Skåne University Hospital, we searched hospital registries for the same KVÅ code. In this search, we identified 64 patients, of whom 18 were excluded since they had undergone another test incorrectly coded as GST or had no available medical records. Altogether 194 patients who had undergone a GST were identified. Of these, 23 had a history of childhood cancer diagnosis or MRI sella pathology and were excluded since they could possibly have disturbances in the HPA axis. In total, 171 children were finally included in the study.

For all children, we registered the following parameters from the medical records: sex; age; pubertal status; height standard deviation score (SDS); weight SDS; BMI SDS; sitting height ratio SDS; mid-parental height (MPH) SDS; birth weight; birth height; small for gestational age (SGA); Insulin-like growth factor 1 (IGF-1) level; basal cortisol level; the use of sex steroids for priming; glucose, growth hormone (GH) and cortisol levels during glucagon test at -30, 0, 30, 60, 90, 120 and 180 min; occurrence and result of magnetic resonance imaging (MRI) sella; treatment with cortisone; levothyroxine; rhGH; gonadotropin-releasing hormone (GnRH) analogue; oestradiol; testosterone and desmopressin; childhood cancer diagnosis; diagnosis of growth hormone deficiency (GHD); indication for test; and centre for testing.

This study was approved by the Swedish Ethical Review Authority (Dnr 2023-05588-01).

### Glucagon stimulation test

The GST started between 8:00 a.m.– 9:00 a.m. at the outpatient clinic. All children were fasting from midnight.

Pubertal status was assessed through physical examination using the Tanner scale. Prepubertal girls were defined as those with a Tanner breast scale of 1, while prepubertal boys were defined as those with a Tanner genital scale of 1. Prepubertal girls > 8 years old and prepubertal boys > 9 years old were considered for priming with sex steroids, where 17-beta-estradiol (T Progynon) would be used: 1 mg/day for weight < 20 kg and 2 mg/day for weight > 20 kg, for 3 constitutive days with the last dose the day before testing. Intravenous access was mostly established the day before the GST, but for some children at the same day. The child was given 30 µg/kg (maximum 1 mg) Glucagon 1 mg/ml as an intramuscular or subcutaneous injection. Plasma glucose, GH and cortisol were measured at -30, 0, 30, 60, 90, 120 and 180 min.

### Biochemical analyses

Plasma cortisol was analysed with the Cortisol II assay standardised against IRMM/IFCC 451 (ID-GC/MS)] from Roche Diagnostics (Mannheim, Germany). The assay is an Electrochemiluminiscence immunoassay (ECLI), which shows an excellent agreement with LC/MSMS measurement of cortisol [17]. The tests were performed at the Department of Clinical Chemistry and Pharmacology in Skåne Regional and University Laboratories (Malmö, Sweden) and at Karolinska University Laboratory (Stockholm, Sweden). The detection limit was 1.5 nmol/L, and the functional detection limit was 3.0 nmol/L. The coefficient of variation (CV) was 3% at 38 nmol/L and 2% at 550 nmol/L. A cut-off level of *≥* 450 nmol/L was considered indicative of a sufficient cortisol secretion using the Roche Cortisol II method, according to a consensus from the Swedish Endocrinological Association, Swedish Society for Clinical Chemistry and Equalis (2017) [13].

Plasma GH was analysed by 3 different methods: Cobas Roche, IDS-iSYS and Immulite 2000 XPi (see supplemental Table 2 for more details). A cut-off level of 6–7 µg/L (depending on method) was used for assessment of a normal GH secretion. For Cobas Roche and Immulite 2000 XPi, values below the measuring range (< 0.05 µg/L) were assigned the value 0.05 µg/L, and for IDS-iSYS values below the measuring range (< 0.02 µg/L) were assigned the value 0.02 µg/L.

Plasma IGF-1 was analysed by 2 different methods: IDS-iSYS and Immulite 2000 XPi (see supplemental Table 2 for more details). The reference interval and values for the 50th percentile depended on age and sex for both methods. The proportion of children with IGF-1 above the 50th percentile is reported in the manuscript, and no absolute numbers are mentioned. For IDS-iSYS IGF-1, values below the measuring range (< 10 µg/L) were assigned the value 10 µg/L, and Immulite 2000XPi values below the measuring range (< 15 µg/L) were assigned the value 15 µg/L.

Plasma glucose was analysed at the paediatric outpatient clinic by point-of-care plasma glucose instruments, values registered as nmol/L.

### Statistical analysis

All statistical analyses were performed using IBM^®^ SPSS^®^ Statistics, version 29. Descriptive data were presented as count, per cent of valid patients and as median (min-max). To compare continuous variables, the Mann-Whitney U test was used, and to compare the distribution of categorical variables, Fisher’s Exact test and Chi-Square test were used. To examine the relationships between cortisol max and different variables, the Spearman’s rho correlation coefficient was calculated. Linear regression analysis was used to examine factors affecting cortisol max at the GST. Confidence intervals (CI) were set to 95%, and P values < 0.05 were considered statistically significant.

## Results

### Characteristics of study participants

In this retrospective study, we investigated cortisol levels in 171 children evaluated with the GST. The children were tested due to short stature (*n* = 162, 94.7%) or due to low growth velocity (*n* = 9, 5.3%).

A summary of clinical characteristics of the included children is presented in Table 1 (median age 7.8 years (range 1.0–18.0), 60 (35.1%) female, 23 (13.5%) pubertal).

**Table 1:** Clinical characteristics for the study population

	*n* (%) or median (min– max)
Number of patients	171 (100)
Female / male	60 (35.1) / 111 (64.9)
Pubertal	23 (13.5)
Priming with sex steroids	50 (29.4)
SGA at birth	34 (21.7)
MRI sella performed	74 (43.3)
Diagnosed with GHD	52 (30.4)
rhGH treatment	85 (49.7)
Reported treatment with inhalation steroids	15 (8.8)
Stimulated peak cortisol < 450 nmol/L	26 (15.2)
IGF-1 above 50th percentile	14 (8.5)
Age, years	7.8 (1.0–18.0)
Weight, SDS	-2.2 (-6.6–2.5)
Height, SDS	-2.8 (-9.0–1.2)
BMI, SDS	-0.2 (-3.6–3.3)
Stimulated peak cortisol, nmol/L	628 (202–1008)
Baseline cortisol, nmol/L	316.5 (84–956)

Values presented as median (min-max) and (%). % = valid percent

BMI: Body Mass Index; GHD: growth hormone deficiency; IGF-I: insulin-like growth factor-1; MRI: magnetic resonance imaging; SDS: standard deviation score; SGA: small for gestational age; rhGH: recombinant human growth hormone

It was known that 15 children (8.8%) might have received treatment with inhaled steroids within the last month prior to the GST.

Of the children included, 131 (76.6%) were tested at the Karolinska University Hospital, Stockholm, Sweden, and 40 (23.4%) were tested at Skåne University Hospital, Malmö, Sweden.

### Description of glucose, GH and cortisol values during glucagon stimulation test

**Fig. 1:**
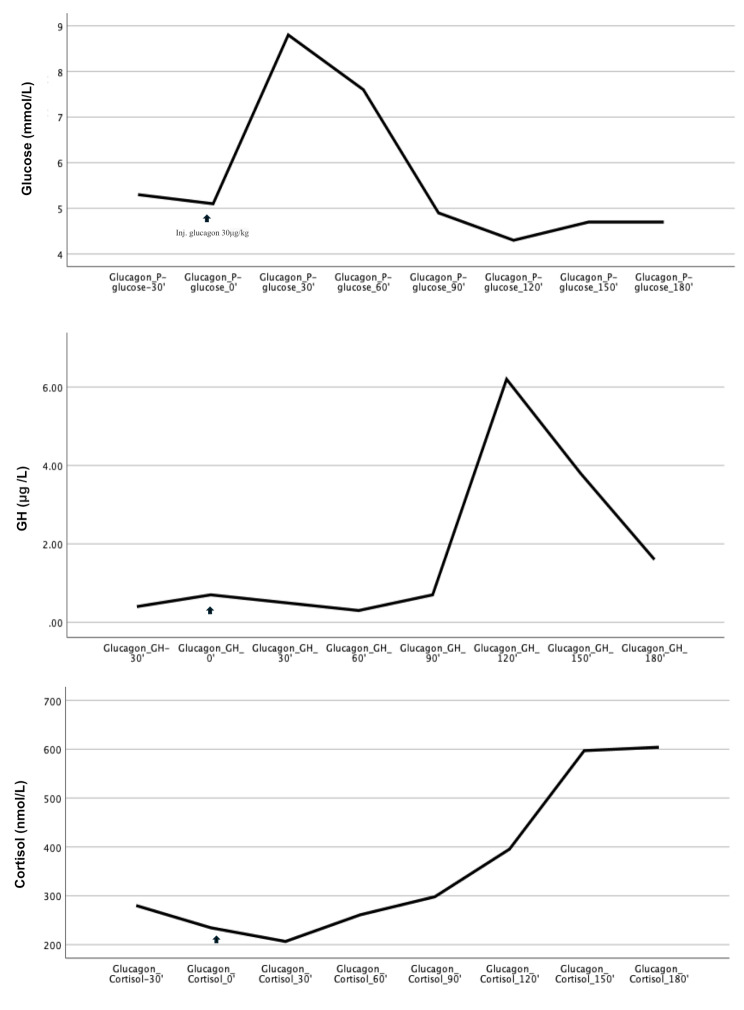
Cortisol, glucose and GH levels (median) at glucagon stimulation test from − 30 min to end of test at 180 min. Arrow representing t = 0 min when the patient was given glucagon 1 mg/ml 30 µg/kg (maximum 1 mg) as an intramuscular or subcutaneous injection GH: Growth hormone

**Fig. 2:**
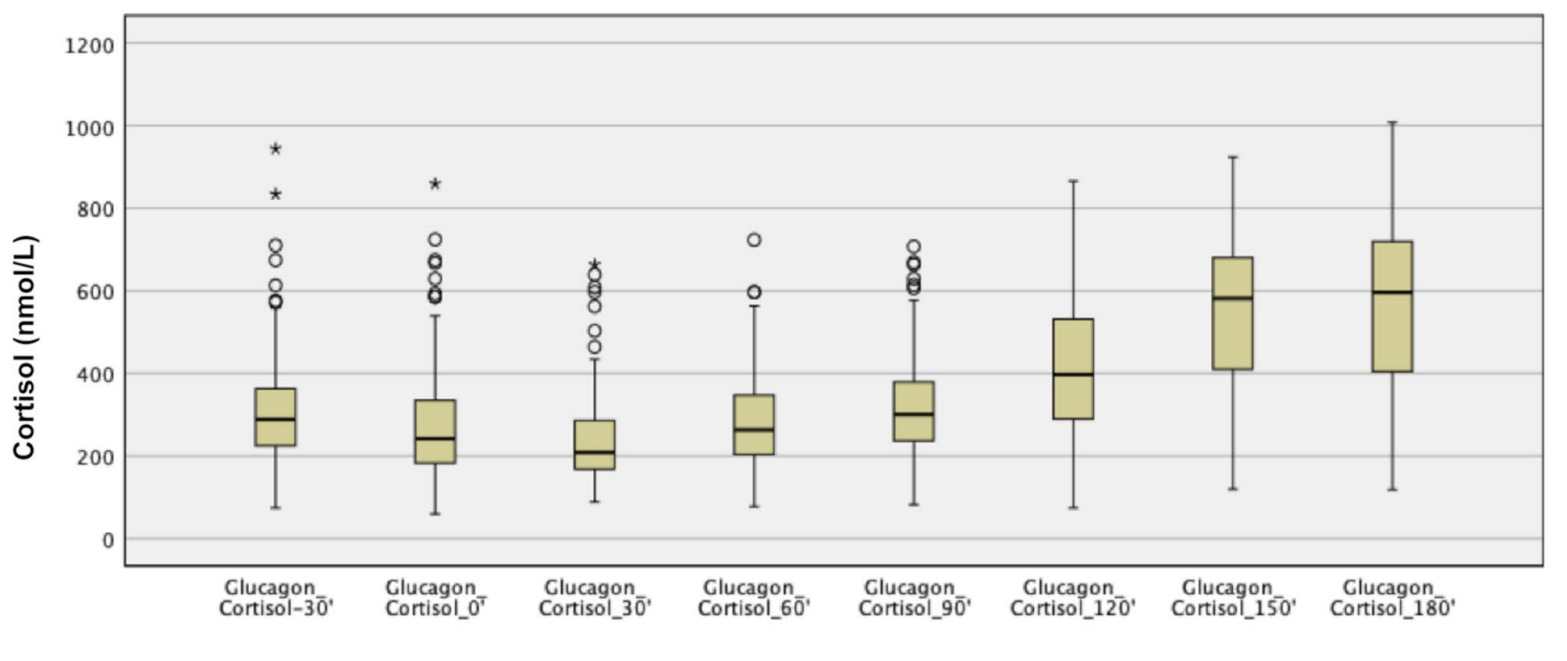
Boxplot illustrating cortisol values (nmol/L) at glucagon stimulation test (GST) from − 30 min to end of test at 180 min

The median cortisol max at GST was 628 nmol/L (range 202–1008 nmol/L). The cortisol levels increased at the end of the GST, after decreasing glucose and GH levels. The median glucose, GH and cortisol values during the GST, from − 30 min to 180 min, are shown in Fig. 1 and in supplemental Fig. 1 (boxplot). In Fig. 2, the cortisol values during the GST are described.

After administration of glucagon, 1182 of 1197 cortisol samples were analysed (98.7%), and no patient had more than one missing cortisol value after glucagon administration. For GH, 1184 of 1197 (98.9%) samples, and for glucose, 1187 of 1197 (99.2%) samples were analysed after administration of glucagon.

### Sex differences

Table 2 summarises clinical characteristics and cortisol levels depending on sex. Girls had higher stimulated peak cortisol at GST (median 667.5 nmol/L vs. 602 nmol/L, *p* = 0.005) compared to boys. For children after start of puberty, girls also had higher peak cortisol at GST than boys (median 672 nmol/L vs. 490 nmol/L, *p* = 0.002). Comparing girls to boys, there was no difference in the frequency of children who may have been treated with inhaled corticosteroids during the month prior to the GST (*p* = 0.882).

**Table 2:** Clinical characteristics for girls and boys

	Girls *n* (%) or median (min– max)	Boys *n* (%) or median (min– max)	*P*-value
Number of patients	60 (35.1)	111 (64.9)	
Pubertal	6 (10.0)	17 (15.3)	0.331^b^
rhGH treatment	29 (48.3)	56 (50.5)	0.792^b^
Reported treatment with inhalation steroids	5 (8.3)	10 (9.0)	0.882^b^
Age, years	7.5 (1.8–14.5)	8.7 (1.0–18.0)	0.102^a^
BMI, SDS	-0.1 (-3.1–3.3)	-0.4 (-3.6–2.8)	0.928^a^
Height, SDS	-2.9 (-4.2–0.8)	-2.8 (-9.0–1.2)	0.537^a^
Stimulated peak cortisol, nmol/L	667.5 (400–995)	602 (202–1008)	0.005^a^

(a) Mann-Whitney U test (b) Chi-square test (c) Fisher’s Exact test. Values presented as median (min-max), and (%). % = valid percent

BMI: Body Mass Index; SDS: standard deviation score; rhGH: recombinant human growth hormone

**Fig. 3:**
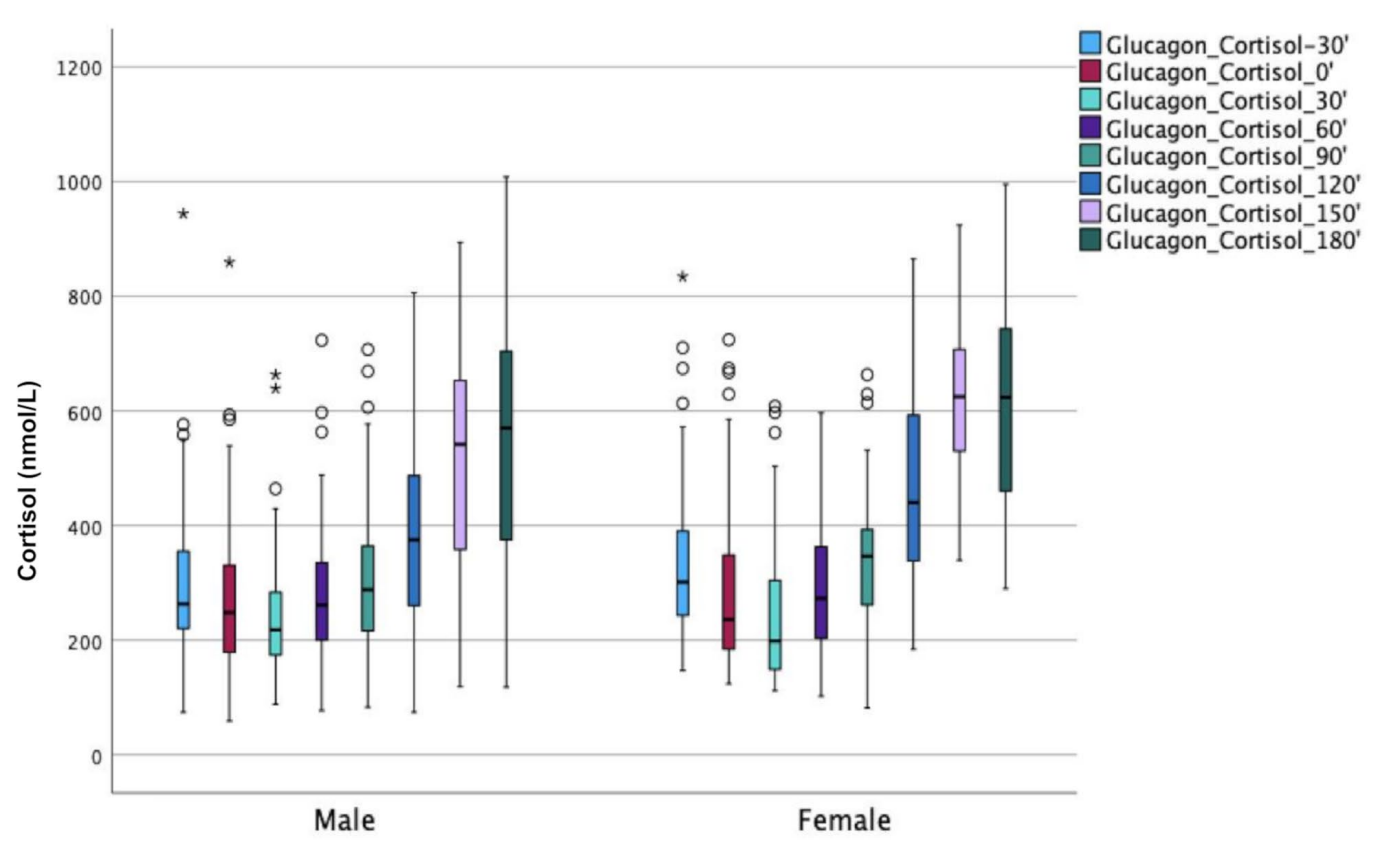
Boxplot illustrating cortisol values (nmol/L) for boys and girls respectively, at glucagon stimulation test from − 30 min to end of test at 180 min

In Fig. 3, the cortisol levels during the GST are shown for boys and girls.

### Cortisol level related to cut-off, 450 nmol/L at glucagon stimulation test– sex-differences

Of all girls, four (6.7%) had a stimulated cortisol max < 450 nmol/L, compared to 22 boys (19.8%). No girls had stimulated cortisol max < 400 nmol/L, whereas 17 boys (15.3%) had cortisol max < 400 nmol/L.

The characteristics of children with cortisol max < 450 (*n* = 26, 15.2%) and *≥* 450 nmol/L (*n* = 145, 84.8%) are described in Table 3. There was a difference in sex distribution, with more girls in the group with cortisol max *≥* 450 nmol/L (*p* = 0.022). There was no difference in use of inhaled steroids between children with peak cortisol < 450 nmol/L vs. children with peak cortisol *≥* 450 nmol/L (*p* = 1.0). Comparison of children with cortisol responses above or below 450 nmol/L revealed no difference in the number who passed or failed to reach the GH cut-off (*p* = 0.199). None of the children had any clinical signs of cortisol deficiency.

**Table 3:** Clinical characteristics for children with glucagon stimulated cortisol < 450 nmol/L and *≥* 450 nmol/L, respectively

	Cortisol < 450nmol/L *n* (%) or median (min– max)	Cortisol *≥* 450nmol/L *n* (%) or median (min– max)	*P*-value	All patients
Number of patients	26 (15.2)	145 (84.8)		171 (100)
Female/male	4 (15.4)/22 (84.6)	56 (38.6)/89 (61.4)	0.022^b^	60 (35.1)/111 (64.9)
Pubertal	6 (23.1)	17 (11.7)	0.126^b^	23 (13.5)
rhGH	10 (38.5)	75 (51.7)	0.213^b^	85 (49.7)
GH response pass/fail	14 (53.8)/12 (46.2)	97 (66.9)/48 (33.1)	0.199^b^	111 (64.9)/60 (35.1)
Reported treatment with inhalation steroids	2 (7.7)	13 (9.0)	1^c^	15 (8.8)
Stimulated peak cortisol (nmol/L)	364.5 (202–444)	663 (450–1008)	< 0.001^a^	628 (202–1008)
Age (years)	8.6 (4.2–18.0)	7.7 (1.0–16.8)	0.141^a^	7.8 (1.0–18.0)
BMI SDS	0.05 (-2.3–2.8)	-0.3 (-3.6–3.3)	0.47^a^	-0.2 (-3.6–3.3)

(a) Mann-Whitney U test (b) Chi-square test (c) Fisher’s Exact test. Values presented as median (min-max), and (%). % = valid percent

BMI: Body Mass Index; GH: Growth hormone; SDS: standard deviation score; rhGH: recombinant human growth hormone

For children with cortisol max < 450 nmol/L, 181 of 182 cortisol samples (98.5%) taken after administration of glucagon were analysed.

### Correlations for cortisol max at glucagon stimulation test

**Fig. 4:**
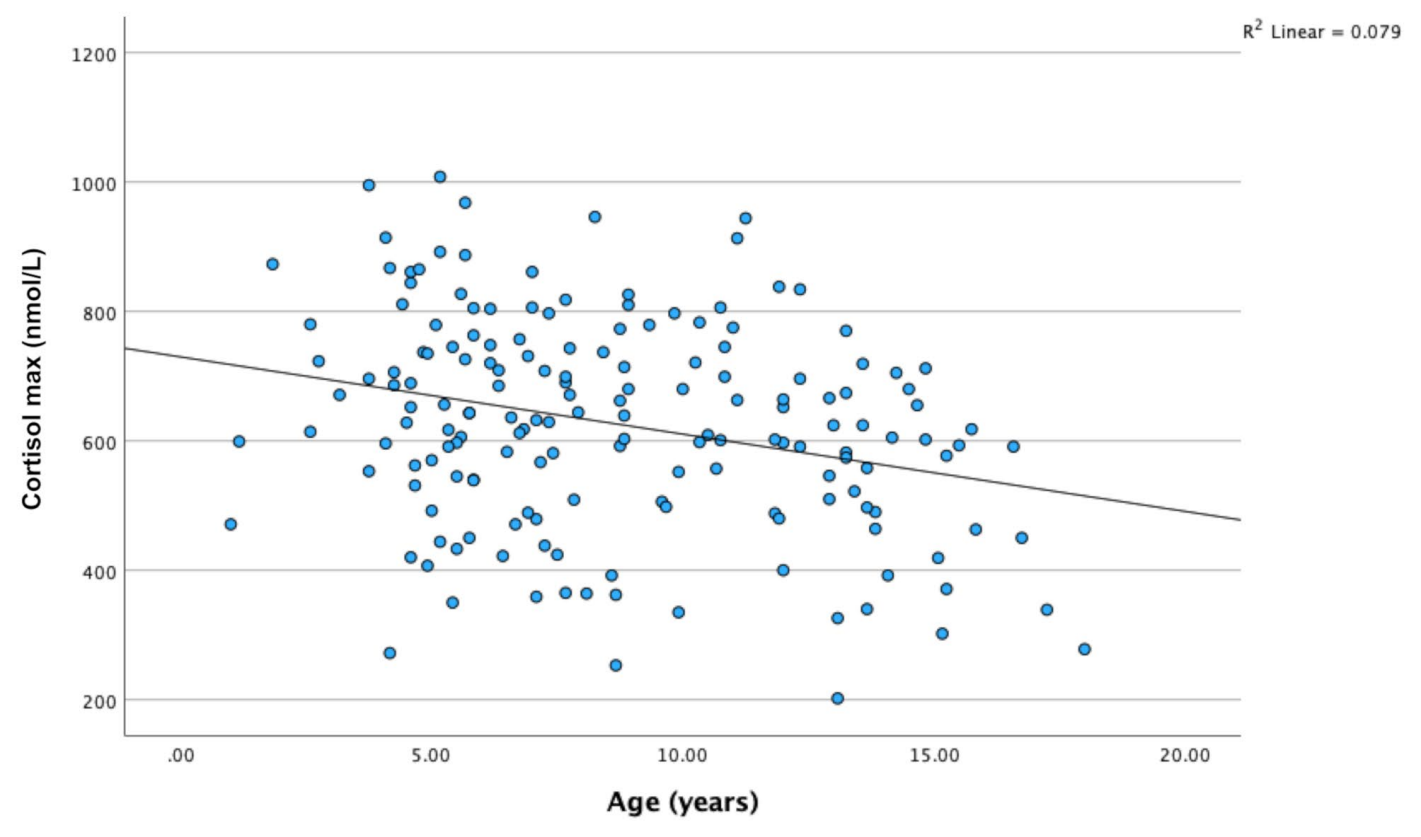
Cortisol max at glucagon stimulation test related to age. Spearman’s rho − 0.26, p = < 0.001, R^2^ = 0.079

Figure 4 describes a negative correlation between cortisol max and age (Fig. 4: Spearman’s rho − 0.26, p = < 0.001, R^2^ = 0.079). There was a correlation between cortisol max and delta glucose (glucose max– glucose min) during the GST (Spearman’s rho 0.19, *p* = 0.014, R^2^ = 0.029). There were no correlations between cortisol max and either glucose min at the GST (*p* = 0.94) or basal cortisol (*p* = 0.17). Additionally, no correlation was found between cortisol max and BMI (*p* = 0.29).

### The impact of sex on cortisol max at glucagon stimulation test– linear regression model

The impact of sex on cortisol max at GST was evaluated with a linear regression model. There was a difference in stimulated cortisol max between the sexes with higher cortisol max in girls than in boys (β (95% CI) 78.1 nmol/L (27.7-128.4) *p* = 0.003), which remained after adjusting for age (β (95% CI) 65.3 (15.9–114.6), *p* = 0.01).

### Puberty and priming with sex steroids

Prepubertal children had higher peak cortisol compared to children after start of puberty (median: 643 nmol/L vs. 577 nmol/L, *p* = 0.005). Priming with sex steroids was given to 50 children (*n* = 170, 29.4%). There was no difference in cortisol max between primed and unprimed children (median 602.5 nmol/ vs. 643 nmol/L, *p* = 0.335, *n* = 170). Information concerning priming was missing for one child.

## Discussion

In this study, we observed that girls evaluated for short stature had higher peak cortisol levels than boys during the GST. Additionally, there was a negative correlation between age and peak cortisol. After adjusting for age, girls still exhibited significantly higher peak cortisol levels compared to boys. Moreover, a higher proportion of boys did not reach the cortisol cut-off (450 nmol/L) used in the study. A total of 145 children (84.8%) achieved a peak cortisol *≥* 450 nmol/L at the GST.

Sex differences of the HPA axis have been reported from the neonatal period to old age [18]. It has been difficult to draw conclusions, since different studies have used both stimulated and unstimulated cortisol levels, and the stressors used to evaluate the HPA axis have been divergent and difficult to compare [18]. In a study by Maliachova et al., 237 children were evaluated for short stature with the GST. The study found significantly higher cortisol peak levels for girls compared to boys, but unlike our study, the sex difference in cortisol levels did not remain for pubertal children [14]. Additionally, Tennenbaum et al. investigated cortisol levels in children (*n* = 190) evaluated with the GST due to short stature and found that peak levels of cortisol were higher among girls and that the rate of cortisol values below cut-off (500 nmol/L) was higher among boys [15]. Weintrob et al. analysed both free serum cortisol and total serum cortisol in 103 children evaluated with the GST and concluded that there was no difference in free serum cortisol between the sexes, but that the total serum cortisol tended to be higher in girls (*p* = 0.05) [12]. In contrast to these results, some studies show no evidence for sex-dependent differences in the cortisol levels of children in pharmacological stress tests of the HPA axis [4, 5, 19]. Other studies have investigated sex differences in salivary cortisol with divergent results [20, 21, 22].

In this study we measured total plasma cortisol and not free cortisol. About 90% of cortisol in plasma is protein bound (80–90% to corticosteroid binding globulin (CBG) and 10–15% to albumin) [12, 23, 24]. In adults, CBG levels seem to be lower in males than females and are negatively correlated with age for both sexes [25]. CBG levels in children have been insufficiently studied. In a previous study, Levine Ross et al. measured CBG levels in 47 children and found a tendency of lower CBG levels in boys than in girls at all ages, although only statistically significant between the ages of 13 and 18 years. However, CBG concentrations did not vary with age in either boys or girls [26]. Based on the results of this study and the findings of Weintrob et al., which suggest that free cortisol values after GST do not vary with age or sex, it can be hypothesized that lower CBG levels in boys may contribute to the observed sex differences in cortisol levels, with higher values in girls [12, 26]. Further studies are required to determine whether analysing free serum cortisol, rather than total cortisol would impact sex-related variations in cortisol levels.

Oestrogens are known to increase the hepatic production of CBG, which affects the bioavailability of cortisol and raises total serum cortisol [24]. This raises a concern of whether the children treated with oestradiol before the GST were more likely to have higher cortisol levels compared to those who were not treated. However, we found no difference in cortisol levels between children who received priming with oestrogen before GST compared to children who did not. This could be related to age differences between primed and unprimed children, but also the short period of time (3 days) the children were treated with oestradiol before the GST.

We found a negative correlation between age and peak cortisol levels at GST, which is in line with the results of previous studies [12, 14, 15, 16]. Weintrob et al. only reported a negative correlation between age and peak total serum cortisol, but this correlation was not seen for free cortisol levels [12]. On the contrary, other studies did not report any correlations between age and cortisol levels at GST [4, 5]. Studies investigating the cortisol peak in children at insulin tolerance test (ITT) and ACTH stimulation test have also shown a negative correlation between peak cortisol and age [27, 28, 29, 30].

In our study, there was a correlation between delta glucose and cortisol max during the GST, but no correlation for the lowest glucose level at the GST. No correlation was seen between cortisol max and BMI SDS. Previous studies have not seen any correlation between BMI SDS/Z scores and the cortisol peak at GST either [14, 15, 16].

The highest cortisol levels during GST were seen at 150 and 180 min, after the glucose and GH levels had decreased from their peak values. The median cortisol values at 150 and 180 min were similar. It is possible that more children would have reached the cortisol cut-off level with a prolonged test. However, a longer test interval would mean a longer fasting period, which is difficult for young children. Most studies evaluating cortisol levels in children at GST analyse cortisol from 0 to 180 min [4, 5, 12, 14, 15]. In one study of 81 children and adults, where GST was evaluated for 210 min, the authors did not see any benefits with a prolonged procedure– the cortisol peak was again seen between 150 and 180 min [31].

In this study, the median peak cortisol value was well above our pre-defined cut-off and 93% of girls and 80% of boys achieved cortisol levels *≥* 450 nmol/L. O’Grady et al. reported that 82% of 223 children evaluated with the ITT had a peak cortisol *≥* 500 nmol/L [27]. We believe that the high percentage of children in our study who achieved cortisol levels *≥* 450 nmol/L demonstrates that the GST is a reliable test of the HPA axis in children, with a lower risk of hypoglycaemia compared to the ITT.

Due to the retrospective design, we were not able to compare the magnitude of the cortisol response of GST to any other provocation test (e.g. ACTH stimulation test). Regardless, it might be advantageous to test the entire HPA axis with GST rather than through direct stimulation of the adrenal gland with the ACTH stimulation test. Given the above, when evaluating the HPA axis in children, the GST may be a viable or even better option than the ACTH stimulation test.

There are weaknesses in our study, mostly related to the retrospective study design. The children included in the study were all evaluated for short stature, which increased their likelihood of having any pituitary abnormality. There were few missing data and < 10% of the children had maximum of one missing cortisol value during the GST. By excluding all children with missing values, there is a risk of selection bias against older children, since it is generally more difficult to collect blood samples in younger children. It was difficult to get reliable information about treatment with inhalation steroids due to a lack of information in the medical records, adherence to treatment, etc., but there was no difference in the reported use of inhaled steroids between any of the groups. Priming with oestradiol for prepubertal girls older than 8 years and boys older than 9 years was part of our testing protocol, which could theoretically influence CBG and cortisol levels. The duration of oestradiol treatment before the GST, however, was brief—only three days. Moreover, the procedures were not completely standardised and were performed by different nurses. Furthermore, different methods were used to analyse GH and IGF-1, and we were only able to evaluate total cortisol, not serum-free cortisol. Glucose was analysed with point-of-care plasma glucose instruments, which may have a lower accuracy.

One strength of this study is that the same cortisol analysis method and GST protocol were used throughout data collection. The size of our study also strengthens our results.

In conclusion, the GST is a reliable test of the HPA axis in children. We found that girls and younger children had higher peak cortisol levels at GST. It is reasonable to ask whether there is a need for sex- and age-dependent reference intervals for peak cortisol values at pharmacological stimulation tests; and whether the analysis of free cortisol would affect the diagnostic utility of the GST relative to the assessment of the HPA axis. More studies are needed, preferably with both the GST and ACTH stimulation test, and to determine sex- and age-dependent reference intervals to avoid under- and over-diagnosing adrenal insufficiency in children.

## Electronic supplementary material

Below is the link to the electronic supplementary material.

Supplementary Material 1

Supplementary Material 2

Supplementary Material 3

## Data Availability

In accordance with Swedish law, information concerning sensitive personal data cannot be shared publicly. Sharing of the data set would generally require a new permission by the Swedish Ethical Review Authority. Contact the corresponding author for request.

## Declarations

Data collection from the medical records were approved by the regional consultation group KVB (quality registry, care information systems, and processing) at Region Skåne, Sweden and by the Unit Manager at Karolinska University Hospital, Stockholm, Sweden. With these permissions, the Swedish Ethical Review Authority postulated that no written informed consent was required for participation in this study.

### Study registration details

This study is not a clinical trial; therefore, study registration details (such as the registry, trial registration number, and registration date) are not applicable.

## Abbreviations

ACTH: adrenocorticotropic hormone
AST: arginine stimulation test
BMI: Body Mass Index
CBG: corticosteroid-binding globulin
CRH: corticotropin-releasing hormone
CV: coefficient of variation
GH: growth hormone
GHD: growth hormone deficiency
GnRH: gonadotropin-releasing hormone
GST: glucagon stimulation test
HPA: hypothalamic-pituitary-adrenal
IGF-1: insulin-like growth factor 1
ITT: insulin tolerance test
KVÅ codes: procedure code numbers
MPH: mid-parental height
MRI: magnetic resonance imaging
SDS: standard deviation score
SGA: small for gestational age

## References

[1] Shulman DI, Palmert MR, Kemp SF. Adrenal insufficiency: still a cause of morbidity and death in childhood. Pediatrics. 2007;119(2):e484–94. 10.1542/peds.2006-1612. 17242136 10.1542/peds.2006-1612

[2] Bornstein SR, Allolio B, Arlt W, Barthel A, Don-Wauchope A, Hammer GD, et al. Diagnosis and treatment of primary adrenal insufficiency: an endocrine society clinical practice guideline. J Clin Endocrinol Metab. 2016;101(2):364–89. 10.1210/jc.2015-1710. 26760044 10.1210/jc.2015-1710 PMC4880116

[3] di Iorgi N, Napoli F, Allegri A, Secco A, Calandra E, Calcagno A, et al. The accuracy of the glucagon test compared to the insulin tolerance test in the diagnosis of adrenal insufficiency in young children with growth hormone deficiency. J Clin Endocrinol Metab. 2010;95(5):2132–9. 10.1210/jc.2009-2697. 20350939 10.1210/jc.2009-2697

[4] Kappy M, Drake A, Gao D, Ratliff R. Assessing adrenal function in primary care settings with a single sample subcutaneous glucagon test. J Pediatr. 2006;149(5):682–6. 10.1016/j.jpeds.2006.07.033. 17095343 10.1016/j.jpeds.2006.07.033

[5] Böttner A, Kratzsch J, Liebermann S, Keller A, Pfaffle R, Kiess W, et al. Comparison of adrenal function tests in children-the glucagon stimulation test allows the simultaneous assessment of adrenal function and growth hormone response in children. J Pediatr Endocrinol Metab. 2005;18(5):433–42. 10.1515/JPEM.2005.18.5.433. 15921172 10.1515/jpem.2005.18.5.433

[6] Fleseriu M, Hashim IA, Karavitaki N, Melmed S, Murad MH, Salvatori R, et al. Hormonal replacement in hypopituitarism in adults: an endocrine society clinical practice guideline. J Clin Endocrinol Metab. 2016;101(11):3888–921. 10.1210/jc.2016-2118. 27736313 10.1210/jc.2016-2118

[7] Arvat E, Maccagno B, Ramunni J, Maccario M, Giordano R, Broglio F, et al. Interaction between glucagon and human corticotropin-releasing hormone or vasopressin on ACTH and cortisol secretion in humans. Eur J Endocrinol. 2000;143(1):99–104. 10.1530/eje.0.1430099. 10870037 10.1530/eje.0.1430099

[8] Lewandowski KC, Lewiński A, Skowrońska-Jóźwiak E, Stasiak M, Horzelski W, Brabant G. Copeptin under glucagon stimulation. Endocrine. 2015;52(2):344–51. 10.1007/s12020-015-0783-7. 26578365 10.1007/s12020-015-0783-7 PMC4824796

[9] Giuffrida FMA, Berger K, Monte L, Oliveira CHMC, Hoff AO, Maciel RMB, et al. Relationship between GH response and glycemic fluctuations in the glucagon stimulation test. Growth Horm IGF Res. 2009;19(1):77–81. 10.1016/j.ghir.2008.06.002. 18678516 10.1016/j.ghir.2008.06.002

[10] Leong KS, Walker AB, Martin I, Wile D, Wilding J, MacFarlane IA. An audit of 500 subcutaneous glucagon stimulation tests to assess growth hormone and ACTH secretion in patients with hypothalamic–pituitary disease. Clin Endocrinol (Oxf). 2001;54(4):463–8. 10.1046/j.1365-2265.2001.01169.x. 11318781 10.1046/j.1365-2265.2001.01169.x

[11] Yalovitsky G, Shaki D, Hershkovitz E, Friger M, Haim A. Comparison of glucagon stimulation test and low dose ACTH test in assessing hypothalamic-pituitary‐adrenal (HPA) axis in children. Clin Endocrinol (Oxf). 2023;98(5):678–81. 10.1111/cen.14887. 36750758 10.1111/cen.14887

[12] Weintrob N, Davidov AS, Becker AS, Israeli G, Oren A, Eyal O. Serum free cortisol during glucagon stimulation test in healthy Short-Statured children and adolescents. Endocr Pract. 2018;24(3):288–93. 10.4158/EP-2017-0132. 29547045 10.4158/EP-2017-0132

[13] Equalis. https://www.equalis.se/media/jcwplzqt/s025_svarsrutiner-för-p-kortisol_1-0.pdf Accessed 3 April 2025.

[14] Maliachova O, Dimitriadou M, Triantafyllou P, Slavakis A, Christoforidis A. Cortisol levels in glucagon stimulation test in children assessed for short stature: clinical and laboratorial correlations. Horm Metab Res. 2019;51(12):798–804. 10.1055/a-1036-6396. 31745940 10.1055/a-1036-6396

[15] Tenenbaum A, Phillip M, de Vries L. The intramuscular glucagon stimulation test does not provide good discrimination between normal and inadequate ACTH reserve when used in the investigation of short healthy children. Horm Res Paediatr. 2014;82(3):194–200. 10.1159/000365190. 25139316 10.1159/000365190

[16] Johnstone HC, Cheetham TD. GH and cortisol response to glucagon administration in short children. Horm Res. 2004;62(1):27–32. 10.1159/000078722. 15166483 10.1159/000078722

[17] Vogeser M, Kratzsch J, Ju Bae Y, Bruegel M, Ceglarek U, Fiers T, et al. Multicenter performance evaluation of a second generation cortisol assay. Clin Chem Lab Med. 2017;55(6):826–35. 10.1515/cclm-2016-0400. 27898397 10.1515/cclm-2016-0400

[18] Panagiotakopoulos L, Neigh GN. Development of the HPA axis: where and when do sex differences manifest? Front Neuroendocrinol. 2014;35(3):285–302. 10.1016/j.yfrne.2014.03.002. 24631756 10.1016/j.yfrne.2014.03.002

[19] Hollanders JJ, van der Voorn B, Rotteveel J, Finken MJJ. Is HPA axis reactivity in childhood gender-specific? A systematic review. Biol Sex Diff. 2017;8(1). 10.1186/s13293-017-0144-8. 10.1186/s13293-017-0144-8 PMC5504848 28693541

[20] van der Voorn B, Hollanders JJ, Ket JCF, Rotteveel J, Finken MJJ. Gender-specific differences in hypothalamus–pituitary–adrenal axis activity during childhood: a systematic review and meta-analysis. Biol Sex Diff. 2017;8(1). 10.1186/s13293-016-0123-5. 10.1186/s13293-016-0123-5 PMC5244584 28116043

[21] Hatzinger M, Brand S, Perren S, Von Wyl A, Stadelmann S, von Klitzing K, et al. In pre-school children, cortisol secretion remains stable over 12 months and is related to psychological functioning and gender. J Psychiatr Res. 2013;47(10):1409–16. 10.1016/j.jpsychires.2013.05.030. 23810195 10.1016/j.jpsychires.2013.05.030

[22] Törnhage C. Reference values for morning salivary cortisol concentrations in healthy school-aged children. J Pediatr Endocrinol Metab. 2002;15(2):197–204. 10.1515/JPEM.2002.15.2.197. 11874185 10.1515/jpem.2002.15.2.197

[23] Arafah BM, Hypothalamic Pituitary Adrenal Function during Critical Illness. Limitations of current assessment methods. J Clin Endocrinol Metab. 2006;91(10):3725–45. 10.1210/jc.2006-0674. 16882746 10.1210/jc.2006-0674

[24] Perogamvros I, Ray DW, Trainer PJ. Regulation of cortisol bioavailability—effects on hormone measurement and action. Nat Rev Endocrinol. 2012;8(12):717–27. 10.1038/nrendo.2012.134. 22890008 10.1038/nrendo.2012.134

[25] Fernandez-Real J, Pugeat M, Grasa M, Broch M, Vendrell J, Brun J, et al. Serum corticosteroid-binding Globulin concentration and insulin resistance syndrome: a population study. J Clin Endocrinol Metab. 2002;87(10):4686–90. 10.1210/jc.2001-011843. 12364459 10.1210/jc.2001-011843

[26] Ross JL, Schulte HM, Gallucci WT, Cutler GB Jr, Loriaux DL, Chrousos GP. Ovine corticotropin-releasing hormone stimulation test in normal children. J Clin Endocrinol Metab. 1986;62(2):390–2. 10.1210/jcem-62-2-390. 3001126 10.1210/jcem-62-2-390

[27] O’Grady MJ, Hensey C, Fallon M, Hoey H, Murphy N, Costigan C, et al. Requirement for age-specific peak cortisol responses to insulin-induced hypoglycaemia in children. Eur J Endocrinol. 2013;169(2):139–45. 10.1530/eje-13-0084. 23672955 10.1530/EJE-13-0084

[28] Crofton PM, Don-Wauchope AC, Bath LE, Kelnar CJH. Cortisol responses to the insulin hypoglycaemia test in children. Horm Res. 2004;61(2):92–7. 10.1159/000075339. 14646394 10.1159/000075339

[29] Lashansky G, Saenger P, Fishman K, Gautier T, Mayes D, Berg G, et al. Normative data for adrenal steroidogenesis in a healthy pediatric population: age- and sex-related changes after adrenocorticotropin stimulation. J Clin Endocrinol Metab. 1991;73(3):674–86. 10.1210/jcem-73-3-674. 1651957 10.1210/jcem-73-3-674

[30] Mushtaq T, Shakur F, Wales J, Wright N. Reliability of the low dose synacthen test in children undergoing pituitary function testing. J Pediatr Endocrinol Metab. 2008;21(12):1129–32. 10.1515/JPEM.2008.21.12.1129. 19189685 10.1515/jpem.2008.21.12.1129

[31] Ach T, Yosra H, Jihen M, Abdelkarim Asma B, Maha K, Molka C, et al. Cortisol cut-points for the glucagon stimulation test in the evaluation of hypothalamic pituitary adrenal axis. Endocr J. 2018;65(9):935–42. 10.1507/endocrj.EJ18-0147. 29952338 10.1507/endocrj.EJ18-0147

