# Supplementary material for: Sex and age differences in cortisol levels during glucagon stimulation test in children
Source: BMC Pediatr. 2025 May 31;25:440. doi: 10.1186/s12887-025-05784-5 (PMC12125804; doi:10.1186/s12887-025-05784-5)
Supplement: Supplementary file 1 — Supplementary Material 1 [file 12887_2025_5784_MOESM1_ESM.docx]

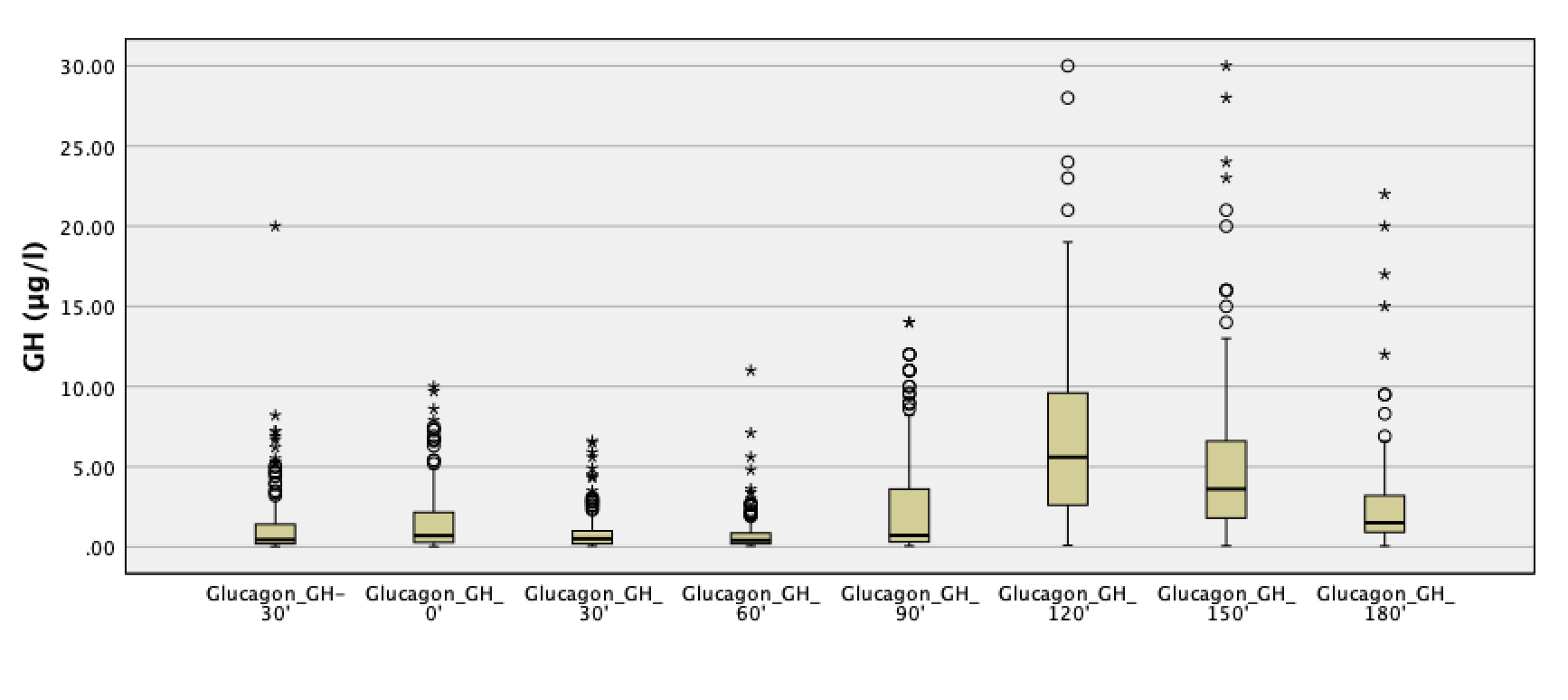

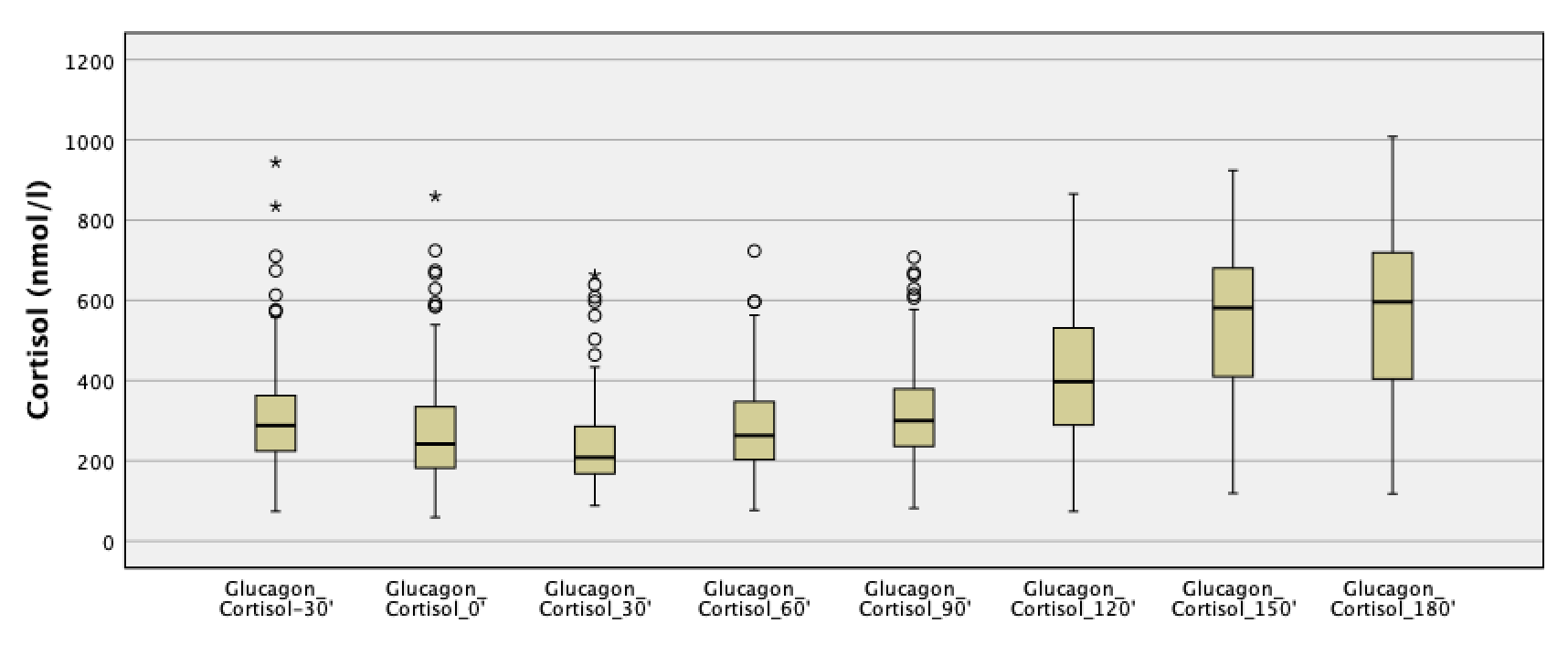

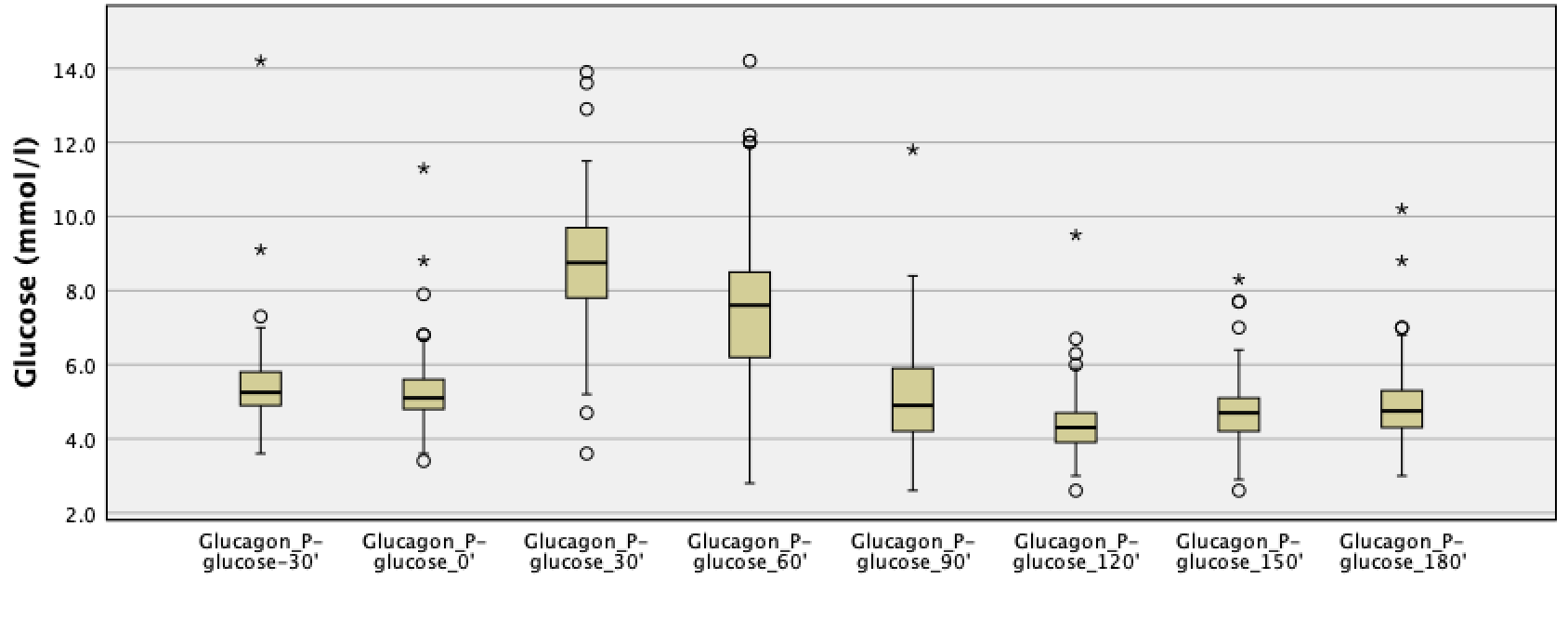


**Cortisol (nmol/L)**

**GH (μg /L)**

**Glucose (mmol/L)**

**Supplemental Fig. 1** Boxplot illustrating cortisol, glucose and GH levels at glucagon stimulation test from -30 minutes to end of test at 180 minutes
