# Supplementary material for: Sex and age differences in cortisol levels during glucagon stimulation test in children
Source: BMC Pediatr. 2025 May 31;25:440. doi: 10.1186/s12887-025-05784-5 (PMC12125804; doi:10.1186/s12887-025-05784-5)
Supplement: Supplementary file 2 — Supplementary Material 2 [file 12887_2025_5784_MOESM2_ESM.docx]

**Supplemental Table 2** Methods and time intervals for biochemical analyses

| Blood sample | Time interval | **Method Malmö** | Detection limit/ Functional detection limit | The coefficient of variation (CV) | **Method Stockholm** | Detection limit/ Functional detection limit | The coefficient of variation (CV) |
| --- | --- | --- | --- | --- | --- | --- | --- |
| Cortisol | 20180101–20231231 | Cobas e601 from Roche Diagnostics (Mannheim, Germany) standardised against IRMM/IFCC 451 (ID-GC/MS). | 1.5 nmol/L /3.0 nmol/L | 3 % at 38 nmol/L and 2 % at 550 nmol/L | Cobas e601 from Roche Diagnostics (Mannheim, Germany) standardised against IRMM/IFCC 451 (ID-GC/MS). | 1.5 nmol/L /3.0 nmol/L | 3% at 38 nmol/L and 2 % at 550 nmol/L |
| GH | 20180101–20201031 | Cobas e601 from Roche Diagnostics (Mannheim, Germany) standardised against WHO 98/574. | 0.03 μg/L/0.05 μg/L | 3 % at 2.0 μg/L and 3 % at 9 μg/L | iSYS from IDS® (Immunodiagnostic Systems Ltd., England), standardised against WHO 98/574. | 0.015 μg/L/ 0.05 μg/L | 10% at 1.5 μg/L and 9% at 10 μg/L |
| GH | 20201101–20210621 | Cobas e601 from Roche Diagnostics (Mannheim, Germany) standardised against WHO 98/574. | 0.03 μg/L/0.05 μg/L | 3 % at 2.0 μg/L and 3 % at 9 μg/L | Immulite 2000XPi from Siemens Healthcare Diagnostics, Gwynedd, UK standardised against WHO 98/574. | Detection limit not given. | 6,5 % at 2,6 μg/L and 4.2 % at 8 μg/L |
| GH | 20210622–20231210 | iSYS from IDS® (Immunodiagnostic Systems Ltd., England), standardised against WHO 98/574. | 0.015 μg/L/ 0.05 μg/L | 10 % at 1,5 μg/L and 9 % at 10 μg/L | Immulite 2000XPi from Siemens Healthcare Diagnostics, Gwynedd, UK standardised against WHO 98/574. | Detection limit not given. | 6,5 % at 2,6 μg/L and 4.2 % at 8 μg/L |
| GH | 20231211–20231231 | Cobas e601 from Roche Diagnostics (Mannheim, Germany) standardised against WHO 98/574. | 0.03 μg/L/0.05 μg/L | 3 % at 2.0 μg/L and 3 % at 9 μg/L | Immulite 2000XPi from Siemens Healthcare Diagnostics, Gwynedd, UK standardised against WHO 98/574. | Detection limit not given. | 6,5 % at 2,6 μg/L and 4.2 % at 8 μg/L |
| IGF-1 | 20180101–20201031 | iSYS from IDS® (Immunodiagnostic Systems Ltd., England) standardised against WHO 02/254. | 10 μg/L/not given | 6% at 63 μg/L, 6% at 260 μg/L, and 6% at 770 μg/L | iSYS from IDS® (Immunodiagnostic Systems Ltd., England) standardised against WHO 02/254. | 10 μg/L/not given | 6 % at 63 μg/L, 6 % at 260 μg/L, and 6 % at 770 μg/L |
| IGF-1 | 20201101–20231231 | iSYS from IDS® (Immunodiagnostic Systems Ltd., England) standardised against WHO 02/254. | 10 μg/L/not given | 6 % at 63 μg/L, 6 % at 260 μg/L, and 6 % at 770 μg/L | Immulite 2000XPi from Siemens Healthcare Diagnostics, Gwynedd,UK, standardised against WHO 02/254. | 13.3 μg /24.9 μg/L | 7.6 % at 56 μg/L, 3.9 % at 200 μg/L and 3.4 % at 621 μg/L |

GH: growth hormone; IGF-1: insulin-like growth factor 1
