# Supplementary material for: Sex and age differences in cortisol levels during glucagon stimulation test in children
Source: BMC Pediatr. 2025 May 31;25:440. doi: 10.1186/s12887-025-05784-5 (PMC12125804; doi:10.1186/s12887-025-05784-5)
Supplement: Supplementary file 3 — Supplementary Material 3 [file 12887_2025_5784_MOESM3_ESM.docx]

**Supplemental Table 1** Summary of studies investigating cortisol levels at glucagon stimulation test (GST) in children

| **First author (year)** | **Aim** | **Subjects** | **Test/ Cortisol cut-off** | **Age** | **Sex** | **Puberty** | **BMI /Weight** | **Basal cortisol** | **Glucose** | **Conclusion** |
| --- | --- | --- | --- | --- | --- | --- | --- | --- | --- | --- |
| Yalovitsky  (2022) | To retrospecitively assess the accuracy of the GST compared to the LD-ACTH test in the diagnosis of adrenal insufficiency. | n=94 (74 males (78.7%))  Mean age (±SD):  10 years ± 3.98 | Glucagon  30 microg/kg, 0-180 min  Suggested cortisol cut- off: 320 nmol/L  (sensitivity 56% specificity 46%)  Currently accepted cortisol cut-off (according to this study): 500 nmol/L | - | - | - | - | - | - | The GST is not an optimal test for diagnosin adrenal insufficiency. |
| Maliachova (2019) | To retrospectively assess cortisol levels in children evaluated for short stature with GST and to correlate the cortisol response to clinical and laboratory data. | n=237 (160 males (67.5%))  Mean age:  9 years (range 1.9 – 16.5)  prepubertal:  n=186 (78.5%) | Glucagon  30 microg/kg, 0-180 min  Cortisol cut-off used in this study: 414 nmol/L | Age: negative correlation to peak cortisol and cortisol AUC. | Girls had higher peak cortisol levels than boys.  Cortisol peak levels in females compared to males:  740.2 ± 201.7 nmol/L vs. 663.3 ± 198.6 nmol/L (recalculated from μg/dl), p = 0.002 | Prepubertal girls had higher peak cortisol than prepubertal boys.  No difference in peak cortisol for pubertal boys and girls. | No association between peak cortisol and BMI Z-Score  Weight: Cortisol AUC was inversely correlated to weight Z-scores (r = − 0.160, p = 0.014). | - | - | The peak cortisol response in GST was age and sex dependent, with higher peak cortisol in younger children and in girls. Also, thinner children had higher cortisol response. |
| Weintrob (2018) | To prospectively assess the serum free cortisol response to the GST in children and determine whether it could predict the GH response to glucagon stimulation. | n=103 (62 males (60.2%))  Median age:  3.9 years (range 0.5 – 14)  Prepubertal:  n = ? | Glucagon  30 microg/kg, 0-180 min  Suggested cortisol cut- off: 436 nmol/L  (sensitivity n/a specificity n/a) | Age: negative correlation to peak total cortisol, but not to serum free cortisol. | Trend for increased total cortisol levels for girls (p 0.05), not for free cortisol. | - | - | - | - | The peak cortisol response in GST was age dependent, with higher peak total cortisol in younger children. There was a non-significant trend for increased total cortisol levels for girls. |
| Tennenbaum (2014) | To prospectively assess the diagnostic value of the GST in evaluating the adrenocortical response in short healthy children. | n=190 (112 males (58.9%))  Mean age:  7.7 ± 4.4 years  Prepubertal:  n=160 (70.8%) | Glucagon  30 microg/kg, 0-180 min  Cortisol cut-off used in this study: 500 nmol/L | Age: negative correlation to peak cortisol.  (When calculated by gender only, significant for boys.) | Girls had higher mean basal cortisol and peak cortisol.  Cortisol peak levels in females compared to males:  741 ± 102 vs. 595 ± 208 nmol/L (p < 0.001).  The proportion of boys with peak cortisol <500 nmol/L was higher compared to girls p<0.001 | Prepubertal children had higher peak cortisol than pubertal patients. | No association between peak cortisol and BMI SDS (not for entire cohort or by gender). | Positive association to peak cortisol. | No association between delta glucose and peak cortisol. | The peak cortisol response in GST was age- and sex-dependent with higher peak cortisol in younger children and in girls. |
| Di Lorgi (2010) | To prospectively assess the accuracy of the GST compared to the ITT in the diagnosis of adrenal insufficiency in children with GHD. | n = 48 (30 males (62.5%)  Median age: 4.2 years ± 1.0 year  Prepubertal: n=48 (100%) | Glucagon  30 microg/kg, 0-180 min  Suggested cortisol cut- off: 403 nmol/L (sensitivity 66.7%, specificity 100%) | - | - | - | - | - | - | The GST is an accurate diagnostic test to validate adrenal function in young children. |
| Kappy  (2006) | To evaluate GST in assessing adrenal gland function. | n=275 (194 males (70%)) (For GST n=215)  Mean Age = ? (range 0.1 – >15 years)  Prepubertal:  n=? | Glucagon  50 microg/kg, 0-150/180 min  Suggested cortisol cut- off: 262 nmol/L (sensitivity n/a specificity 95%) | Age: No age difference in cortisol response. | No sex difference in cortisol response. | - | - | No correlation in change of cortisol from basal cortisol to peak cortisol. | - | The cortisol response in GST was not correlated to sex or age.  The GST had 90.5% concordance with ACTH test in evaluating the HPA-axis. |
| Böttner  (2005) | To evaluate GST in assessing adrenal gland function. | n=290 (191 male (65.9%)) (For GST n=120)  Mean age:  10.1 ± 5.0 years  Prepubertal:  n=? | Glucagon  50 microg/kg, 0-180 min  Suggested cortisol cut-off: 450 nmol/L  (sensitivity 88.5%, specificity 86.8%) | Age: No age difference in cortisol response. | No sex difference in cortisol response.  Cortisol peak levels in females compared to males:  671.6 ± 26.0 vs. 657 ± 24.9 nmol/L (p < ?). | - | - | - | No association between peak cortisol and glucose levels. | The cortisol response in GST was not correlated to glucose levels, sex or age.  GST is a reliable test for assessment of adrenal gland function. |
| Johnstone (2004) | To retrospectively  asses if there is a link between GH status and blood glucose response at GST and to identify determinants of peak GH and peak cortisol. | n=87 (? males)  Median age;  8.13 years (range 1.2 – 20.5)  Prepubertal:  n=? | Glucagon 1 mg, 0-240 min  Cortisol cut-off used in this study: 500 nmol/L | Age: negative correlation to peak cortisol (n=41, GST in short, “normal” children).  (although not for prepubertal children only). | - | - | No association between peak cortisol and BMI SDS.  Weight: negative correlation to peak cortisol, although not for prepubertal children. | - | No association between glucose (baseline, peak and delta) and cortisol peak. | The peak cortisol response in GST was age dependent, with higher cortisol peak in younger children. |
| Current study  Borghammar | To retrospectively assess cortisol levels at GST, and to evaluate how clinical parameters correlates to cortisol levels, in children with short stature. | n=171 (111 male (64.9%))  Median age:  7.8 years (range 1.0 – 18.0)  Prepubertal:  n=148 (86.5%) | Glucagon 30μg/kg, 0-180 min  Cortisol cut-off used in this study: 450 nmol/L | Age: negative correlation to peak total cortisol. | Girls had higher peak cortisol (both for all children and for pubertal children).  Cortisol peak levels (median) in females compared to males:  667.5 nmol/L (range 400 – 995) vs. 602 nmol/L (range 202 – 1008), p = 0.005  Proportion of boys with cortisol <450 nmol/L was higher (p = 0.022).  Linear regression model; difference in stimulated cortisol max between the sexes, which remained after adjusting for age. | Prepubertal children had higher peak cortisol than children after start of puberty.  Cortisol peak levels (median) prepubertal compared to pubertal:  643 nmol/L vs. 577 nmol/L, p=0.005 | No association between peak cortisol and BMI SDS. | No association between basal cortisol and max cortisol. | No association between glucose min and max cortisol.  Positive correlation between delta glucose and cortisol max. | The cortisol peak at GST was related to sex and age. Girls and younger children had higher cortisol max at GST. |

*Abbreviations*: AUC: area under the curve; GH: growth hormone; GHD: growth hormone deficiency; GST: glucagon stimulation test; HPA-axis: hypothalamic-pituitary-adrenal-axis; ITT: Insulin tolerance test; LD-ACTH: low dose ACTH-test; min: minutes.
